# Range of metastrongylids (superfamily Metastrongyloidea) of public health and veterinary concern present in livers of the endemic lizard *Gallotia* *galloti* of Tenerife, Canary Islands, Spain

**DOI:** 10.1186/s13071-023-05653-z

**Published:** 2023-03-01

**Authors:** Elena Izquierdo-Rodriguez, Lucia Anettová, Kristýna Hrazdilová, Pilar Foronda, David Modrý

**Affiliations:** 1 Instituto Universitario de Enfermedades Tropicales y Salud Pública de Canarias, Universidad de La Laguna, Santa Cruz de Tenerife, Spain; 2 Departamento de Obstetricia y Ginecología, Pediatría, Medicina Preventiva y Salud Pública, Toxicología, Medicina Legal y Forense y Parasitología, Facultad de Farmacia, Universidad de La Lagunas, Santa Cruz de Tenerife, Spain; 3 Department of Botany and Zoology, Faculty of Science, Masaryk University, Brno, Czech Republic; 4 Department of Chemistry and Biochemistry, Faculty of AgriSciences, Mendel University in Brno, Zemědělská 1665/1, Brno, Czech Republic; 5 Faculty of Medicine in Pilsen, Biomedical Center, Charles University, Alej Svobody 1655/76, Plzeň, Czech Republic; 6 Institute of Parasitology, Biology Center of Czech Academy of Sciences, Prague, Czech Republic; 7 Department of Veterinary Sciences, Faculty of Agrobiology, Food and Natural Resources (CINeZ), Czech University of Life Sciences, Prague, Czech Republic

**Keywords:** *Gallotiagalloti*, *Angiostrongyluscantonensis*, *Angiostrongylusvasorum*, *Aelurostrongylusabstrusus*, *Crenosomastriatum*, Multiplex PCR

## Abstract

**Background:**

Endemic lizards of the genus *Gallotia* are of high ecological value to the terrestrial ecosystem of the archipelago of the Canary Islands, being potent seed spreaders as well as an important component of the diet of other vertebrates. The endemic lizard *Gallotia* *galloti* in Tenerife has recently been reported to be a paratenic host of *Angiostrongylus* *cantonensis*, an invasive metastrongylid with zoonotic potential that is associated with rats as definitive hosts. However, microscopic examination of *G.* *galloti* tissue samples also revealed the presence of other metastrongylid larvae inside granulomas on the liver of this reptile. The aim of this study was to investigate the presence of helminths other than *A.* *cantonensis* in tissues of *G.* *galloti* from Tenerife.

**Methods:**

A multiplex-nested PCR targeting the internal transcribed spacer 1 was designed that enabled the species-specific detection of *A.* *cantonensis*, *Angiostrongylus* *vasorum*, *Aelurostrongylus* *abstrusus*, *Crenosoma* *striatu*m and *Crenosoma* *vulpis*. Liver samples from 39 *G.* *galloti* were analysed.

**Results:**

Five metastrongylids were detected: *A.* *cantonensis* (15.4% of samples analysed), *A.* *vasorum* (5.1%), *Ae.* *abstrusus* (30.8%), *C.* *striatum* (30.8%) and undetermined metastrongylid sequences (12.8%). Co-infection was highly prevalent among the lizards which tested positive.

**Conclusions:**

The study provides a new specific tool for the simultaneous detection of a range of metastrongylids of veterinary importance as well as new data on the circulation of metastrongylids in an ecosystem dominated by lizards.

**Graphical Abstract:**

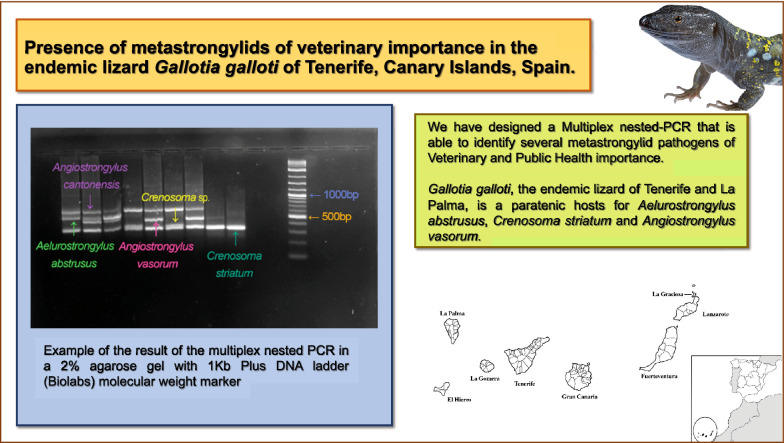

## Background

Nematodes of the superfamily Metastrongyloidea are characterised by an indirect life-cycle involving gastropods (or less frequently annelids) as intermediate hosts in which third-stage (L3) larvae develop [1]. Although the presence of a gastropod intermediate host is essential in the life-cycle of these nematodes, the definitive hosts are commonly infected by ingestion of a vertebrate paratenic host, such as amphibians, reptiles, rodents and shrews. Moreover, an invertebrate transport host can be involved, such as crustaceans or insects in the case of the parasitic nematode *Angiostrongylus* *cantonensis* [43].

The survival of L3 larvae in vertebrate tissues and the importance of the paratenic hosts for the circulation of metastrongylid parasites across ecosystems is evident from studies on the most studied nematode species. *Angiostrongylus* *cantonensis*, the rat lungworm, has been found infecting fish, frogs, toads, snakes, lizards and monitor lizards [2, 4, 34, 39, 44], and the ingestion of such poikilothermic hosts is responsible for a proportion of human infections [17, 25]. The case *of* *Angiostrongylus* *vasorum* parasitising canids, the common frog *Rana* *temporaria* and domestic chicken were proven to be paratenic hosts under experimental conditions; however, there are no records of naturally infected paratenic hosts, probably due to the paucity of detection methods [6, 33]. In a number of different studies, *Aelurostrongylus* *abstrusus*, the cat lungworm, was experimentally transmitted to mice, chickens, ducklings, frogs, toads, snakes and lizards [1, 13, 16, 18, 27], but only the striped field mouse *Apodemus* *agrarius* has been found to be naturally infected [20]. For many metastrongyloids, the existence of paratenic hosts has either not been studied or not confirmed, such as, for example, *Crenosoma* *striatum* and *Crenosoma* *vulpis*, lungworm of hedgehogs and canids, respectively.

The recent emergence of zoonotic *A.* *cantonensis* in Tenerife, the largest island of the Canary Islands, has received much attention due to the proximity of the island to continental Europe. The species has been detected in rats and molluscs [14, 29], as well as in a paratenic host, the endemic lizard *Gallotia* *galloti*[2]. Among other metastrongylids recorded in the Macaronesian archipelago, *C.* *striatum* is commonly found in the Algerian hedgehog *Atelerix* *algirus* of Lanzarote [40], larvae from *A.* *vasorum* and *Ae.* *abstrusus* have been morphologically identified in molluscs from Tenerife [41] and a case of ocular affection for *Gurltia* *paralysans* was reported in a cat of unknown origin in Tenerife [42].

Our recent study empirically demonstrated the presence of metastrongylid larvae other than those of *A.* *cantonensis* in liver samples of *G.* *galloti* [2]. The aim of the present study was to develop the appropriate molecular tool and to investigate the diversity of metastrongylid helminths exploiting endemic lizards of Tenerife as paratenic hosts.

## Methods

### Sample preparation

Thirty-six liver samples from the *G.* *galloti* lizards previously studied by Anettová et al. [2] were used in the present investigation. Briefly, specimens of *G.* *galloti* were captured in Tegueste (Tenerife, Canary Islands, Spain) and euthanised at the Instituto Universitario de Enfermedades Tropicales y Salud Pública de Canarias (IUETSPC). Liver samples were obtained during dissection and preserved in absolute ethanol for molecular analysis. DNA was isolated from the tissue samples using the Qiagen DNEasy Blood & Tissue Kit (Hilden, Germany) with the following modifications: 25 μl of proteinase K and lyse phase extended to overnight.

### Molecular techniques

A nested PCR approach that provided higher sensitivity and specificity was chosen for the detection of *A.* *cantonensis*, *A.* *vasorum*, *Ae.* *abstrusus*, *C.* *striatum* and *C.* *vulpis*. Universal primers amplifying the entire internal transcribed spacer 1 (ITS1) region of all the metastrongylid nematodes included in this study were used for the first round of PCR [38]. For the second round, species-specific primers were designed based on multiple sequence alignment of the ITS1 of *A.* *cantonensis*, *A.* *vasorum*, *Ae.* *abstrusus*, *C.* *striatum* and *C.* *vulpis* using sequences available in GenBank and Geneious Prime® version 2019.2.1 software [24]. To facilitate the identification of products following gel electrophoresis, the size of the product for each targeted species differs by > 40 nt (Table 1). Both rounds of PCRs were performed using the Qiagen Multiplex PCR plus Kit under the following conditions: 95 ºC for 15 min to enable the Hotstart activation, followed by 35 cycles of 94 ºC for 30 s, 57 ºC for 90 s and 72 ºC for 90 s, with a final step of 72 ºC for 10 min. The reaction was performed in a total volume of 25 μl, containing 2 μM of each primer, 12.5 μl of Multiplex PCR Master Mix and 1 μl of DNA template. The specificity of the technique was confirmed by using DNA of helminths from each species with all the primers separately and the same multiplex set-up. Amplified products of the multiplex PCR were visualised in 2% agarose gel, at 75 V for at least 90 min, and the separated bands were later purified using the Gel/PCR DNA Fragments Extraction Kit (Geneaid Biotech Ltd., New Taipei City, Taiwan) and sent for capillary sequencing using the amplification primers to Macrogen Europe BV (Amsterdam, The Netherlands). The obtained sequences were assembled and edited using the Geneious Prime® 2019.2.1 software [24] and identified by BLASTn analysis of the NCBI GenBank database. All unique sequences were deposited into GenBank under accession numbers OP210306-11.

**Table 1:** Primers designed for the multiplex-nested PCR analysis and the molecular weights of the resulting products

PCR Round	Primer	Sequence (5′–3′)	Product size (bp)^a^	Species^a^
1	ITS1_F1674	GTCGTAACAAGGTATCTGTAGGTG		
	ITS1_58SR4	TAGCTGCGTTTTTCATCGATA		
2	ITS1_Canto_F3	AACAACTAGCATCATCTACGTC	642	*Angiostrongylus* *cantonensis*
	ITS1_Canto_R1	CATCCTGTGTATCTCGTTCC		
	ITS1_Aeluro_F1	GCTTTGATCAACGAGAAACC	537	*Aelurostronylus* *abstrusus*
	ITS1_Aeluro_R2	CATACGTGCACAGTATAATCTC		
	ITS1_Vasor_F1	CTCATCGTCATCATCGTTATAG	492	*Angiostrongylus* *vasorum*
	ITS1_Vasor_R1	ACCATATTCAGTAGTCATTGTC		
	ITS1_Creno_s_R2	GTACCACGTAACACACGA	377	*Crenosoma* *striatum*
	ITS1_Creno_F2	TCTGGAATTTTTGTGGATTGG		
	ITS1_Creno_v_R1	GCTACTTATCAAGTAAGCTAGC	299	*Crenosoma* *vulpis*

*ITS1* Internal transcribed spacer 1

^a^Detection method for *C.* *striatum* and *C.* *vulpis* share the forward primer

## Results

**Fig. 1:**
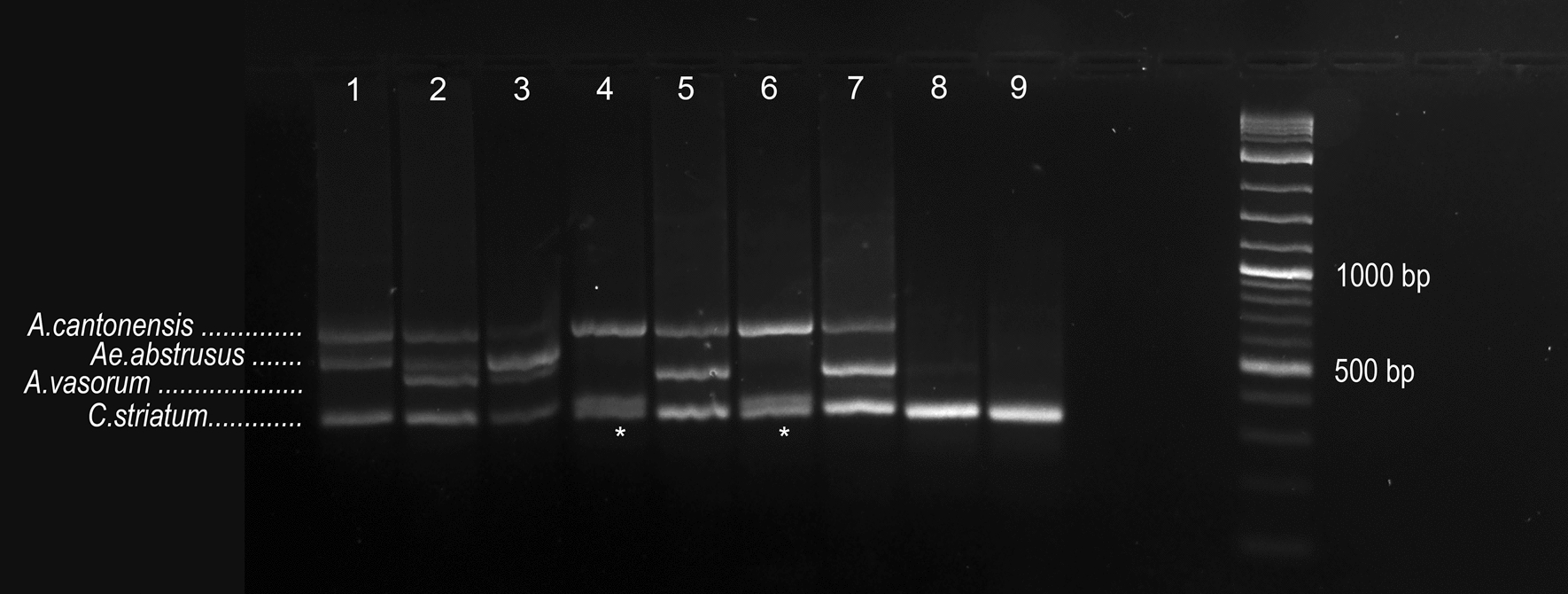
Representative result of the multiplex-nested PCR of DNA-spiked samples showing the identifying/differentiation power of the assay in a 2% agarose gel with 1-kb Plus DNA ladder molecular weight marker (New England Biolabs, Ipswich, MA, USA). In terms of size of the respective products, the 642-bp band represents *Angiostrongylus* *cantonensis*, the 537-bp band represents *Aelurostronylus* *abstrusus, *the 392-bp band represents *Angiostrongylus* *vasorum* and the 377-bp band represents *Crenosoma* *striatum*; in the case of unidentified metastrongylid, an additional band around 300 bp appeared (lanes* 4*,* 6*). Lanes:* 1* *A.* *cantonensis* + *Ae.* *abstrusus* + *C.* *striatum*;* 2* *A.* *cantonensis* + *Ae.* *abstrusus* + *A.* *vasorum* + *C.* *striatum*;* 3* *Ae.* *abstrusus* + *A.* *vasorum* + *C.* *striatum*,* 4*,* 6* *A.* *cantonensis* + unidentified metastrongylid;* 5*,* 7* *A.* *cantonensis* + *A.* *vasorum* + *C.* *striatum*;* 8*,* 9* *C.* *striatum* only

**Fig. 2:**
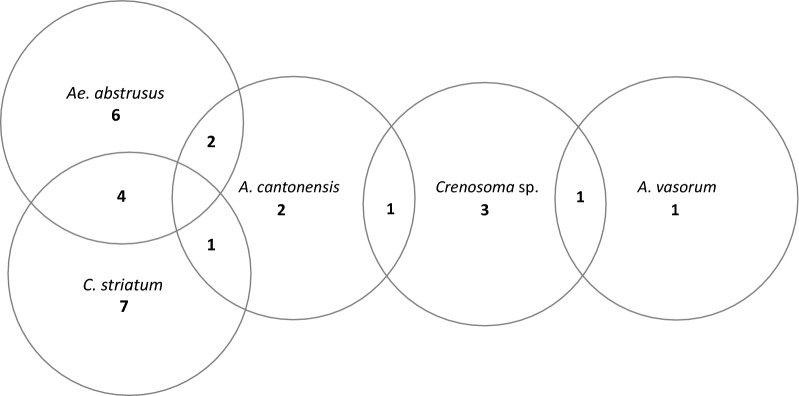
Diagrammatic presentation of co-occurrence of detected species of metastrongylids as detected by the multiplex-nested PCR in liver samples of *Gallotia* *galloti*

The multiplex-nested PCR confirmed the presence of DNA of four of the five helminth species investigated. Sequencing of the products of the expected sizes revealed, in addition to the already known presence of *A.* *cantonensis* in *G.* *galloti* livers, two samples positive for the DNA of *A.* *vasorum* (5.6%) and 12 samples (33.3%) positive for *Ae.* *abstrusus* DNA; 12 samples (33.3%) presented the DNA of *C.* *striatum*. A PCR product of the size corresponding to that in the *C.* *vulpis* assay was not detected in any of the tested samples, but an additional band slightly above the *C.* *striatum* PCR product was observed in five samples (13.8%) (Fig. 1). BLAST analyses of its sequence showed MG878893-4 (assigned as *Crenosoma* sp. in GenBank) as the most closely related sequences with 94.2% and 93.2% similarity, both detected in slugs in Germany [26]. In the case of *A.* *cantonensis*, six samples tested positive by the multiplex PCR, in comparison of the nine detected in the very same set of 36 livers by quantitative PCR [2]. In total, 27 samples were positive for at least one metastrongylid nematode (75%). Co-infections were highly prevalent, and 14 (38.9%) of the total lizard samples analysed tested positive for more than one helminth (Fig. 2).

## Discussion

Reptiles are known to serve as paratenic hosts of *A.* *cantonensis* (Radomyos et al. 1994, [2] as well as of other metastrongylid nematodes and can be a source of infection in wild or domestic animals or humans when eaten raw or undercooked [17, 23].

Trophic transmission is a common route of parasitic helminths’ circulation in the environment. These parasites often reproduce in large vertebrates, which allow them to increase their growth capacity as well as providing longer lifespans. Many larger vertebrates are predators occupying high tropic positions and therefore have limited direct contact with free-living larval stages of helminths or with their eggs. Thus, the nematodes infecting such definitive hosts tend to have an additional host in their life-cycle. The existence of paratenic hosts within one parasite life-cycle not only increases the survival of its larval stages, but also facilitates its transmission into these hosts at higher tropic levels [35]. Paratenesis, the transmission of larvae from a paratenic host to other hosts, is a key ecological adaptation in the transmission of metastrongylids to carnivorous mammals, as they are unlikely to feed on gastropods but commonly hunt other animals, such as rodents or reptiles [1].

Reptiles are important components of the food webs in many ecosystems and, as such, they are function as paratenic hosts in the life-cycles of various helmiths. As examples, Australian snakes and dwarf monitor lizards are hosts for physalopterid nematodes [21, 22]; Nile monitor lizards harbour species of *Dracunculus* [7]; *Trichinella* *papuae* and *T.* *zimbabwensis* have been found infecting reptile species in equatorial regions [37]; *Anisakis* sp. type I is able to infect green turtles (*Chelonia* *mydas*) and crocodiles are also believed to be paratenic hosts of this nematode [8, 28]; water snakes (*Nerodia* *sipedon*) have been reported harbouring species of *Eustrongylides* [9]; and Monitor lizards are a paratenic host of *A.* *cantonensis*, being responsible for outbreaks of eosinophilic meningitis in humans following the ingestion of organs of these large saurians [34].

Metastrongylid larvae are expected to be found mostly in liver tissue in paratenic hosts and, to a lesser extent, in muscles and connective tissues [10, 20, 31]. Based on a field study on *Varanus* *bengalensis* infected with *A.* *cantonensis* (Radomyos et al. 1994), we decided to use liver tissue for our search of metastrongylids using a refined molecular method, as the concentration of larvae and the probability of detection is believed to be the highest in this tissue. Since the liver has a special immune status and is one of the organs most frequently infected by parasites due to its ability to preferentially induce tolerance over immunity, the liver may be utilised by parasites in general to evade host immunity [11]. In the present study, we not only confirmed that the endemic lizard *G.* *galloti* serves as paratenic host of various metastrongylids, but we also reported a novel sensitive and specific diagnostic tool that distinguishes a range of metastrongylid nematodes.

To our knowledge, this is the first study to demonstrate the presence of well-identified larvae of metastrongylids other than *A.* *cantonensis* in tissues of naturally infected reptiles. In our study, 75% of *Gallotia* lizards were positive for at least one metastrongylid nematode, and co-infections were common. These results suggest the high importance of these hosts in the circulation of metastrongyloids. Interestingly, sequence analyses also proved the presence of species whose presence, until this investigation, was unknown for the island of Tenerife or the Canary archipelago as such, thereby demonstrating the value of reptiles as sentinels in the active surveillance of metastrongyloids of medical and veterinary importance.

The common presence of *A.* *abstrusus* in Tenerife lizards (one third of the lizards examined were positive for *A.* *abstrusus* DNA) contrasts with the lack of data on infection on cats, the definitive hosts of this nematode, in Tenerife. The cats of the archipelago have been recorded to prey on *Gallotia* species [30], and colonies of feral cats should be investigated to determine the real extent of this parasite in the island. Local veterinary practitioners should take aelurostrongyliasis into consideration when a cat presents respiratory disease, especially in cases where the animal is allowed to hunt wildlife freely.

We also report the presence of the hedgehog parasite *C.* *striatum* on Tenerife for the first time. Its presence has been suspected due to the high abundance of the Algerian hedgehog *A.* *algirus* in the island and a previous record of *C.* *striatum* in the archipelago [3, 40]. Our results extend current knowledge on the life-cycle of *C.* *striatum*, suggesting an involvement of the vertebrate paratenic host. The diet of *A.* *algirus* in the Canary Islands has not been properly studied, but studies in continental Africa show that it consists mainly of arthropods, although availability of prey is a key factor determining diet selection, with reptile remnants occasionally found in their faeces [12, 32]. Therefore, predation of *Gallotia* lizards cannot be excluded as a source of infection by *C.* *striatum*, especially in the terrestrial ecosystem of the Canary Islands which is relatively poor in insects.

Among the two parasites of canids included in this study, *C.* *vulpes* was absent, but *A.* *vasorum* was detected in two lizards examined. Our data corroborate previous experimental infections of frogs and chicken [6, 33] and extend the spectrum of paratenic host of *A.* *vasorum*. Larvae morphologically identified as *A.* *vasorum* were previously reported from Tenerife molluscs [41]; however, clinical infections of dogs by *A.* *vasorum* have not been reported in Tenerife to date and deserve further attention. Interestingly, our data suggest the presence of an undetermined metastrongylid species infecting *G.* *galloti*, but further genetic, and morphological analyse are needed on this helminth, as only a partial region of the ITS1 gene was amplified during this study.

Undoubtedly, the ecosystem of the Canary Islands is characterised by a dominance of reptiles in its terrestrial fauna, as *Gallotia* species are found inhabiting every ecosystem between 0 and 3000 m a.s.l. [36]. The high population densities and omnivorism of *G.* *galloti* probably underlie the high prevalence of parasites observed. However, it is probable that reptiles also play an important role in the transmission cycles of metastrongyloids in more complex continental ecosystems. Specifically in Mediterranean regions of Europe, where peri-domestic species of reptiles reach very high abundances, these hosts are most probably source of infection by metastrongylids of genera *Aelurostrongylus* and *Troglostrongylus* in cats.

## Conclusions

The multiplex-nested PCR reported here is a highly specific tool that enables detection of co-infections, which is an improvement on the traditional methods of detection of metastrongylids that rely on morphology and generic PCRs [15, 41]. This method can be broadly applied and allows the determination of metastrongyloid species of veterinary and public health importance in new areas and hosts. Apart from its specificity and sensitivity, the design of this new multiplex-nested PCR allows the identification of the studied nematodes without sequencing, which makes it a valuable tool, especially when large sample sets need to be screened. This approach can also be easily adapted to the pathogens prevalent in a specific region; for example primers ITS1_Troglo_F1 (5′-CGACATGTGATCTGTTGTGA-3′) and ITS1_Troglo_R4 (5′-TACATTTGCATGTACATCCAC-3′) have been designed for the detection of *Troglostrongylus* *brevior*, with a product size of 579 bp. However, the approach cannot be used to distinguish between *A.* *cantonensis* and *A.* *mackerrasae* and further analysis should be done to determine the identity of the amplified fragment in areas of co-existence in Australia [5]. Also, for clear differentiation of the resulting bands, the second-round PCR can be split into two independent runs targeting fewer species. Additionally, the technique could be applied in wildlife samples to evaluate hotspots of transmissions of metastrongylids, which is especially interesting in the case of zoonotic *A.* *cantonensis* as identification of foci of eosinophilic meningitis could aid practitioners to identify the causative agent. Overall, this new approach for the detection of metastrongylid parasites constitutes an improvement from traditional methods of larval identification due to its high sensitivity, its ability to detect co-infections, its capacity to provide results quickly with little manipulation of the samples and its application to different tissues, all leading to the identification of new intermediate and paratenic hosts.

## Data Availability

Not applicable.

## Abbreviations

ITS1: Internal transcribed spacer 1
L3: Third-stage larva

## References

[1] Anderson RC (2000). Nematode parasites of vertebrates: their development and transmission.

[2] Anettová L, Izquierdo-Rodriguez E, Foronda P, Baláž V, Novotný L, Modrý D (2022). Endemic lizards *Gallotia*
*galloti* are paratenic hosts in the life cycle of invasive *Angiostrongylus*
*cantonensis* in Tenerife, Spain. Parasitology.

[3] Arechavaleta M, Rodríguez S, Zurita N, García A (2010) Lista de Especies Silvestres de Canarias. Hongos, Plantas y Animales Terrestres. Santa Cruz de Tenerife: Gobierno de Canarias. 2021 (in Spanish).

[4] Asato R, Sato Y, Otsuru M (1978). The occurrence of *Angiostrongylus*
*cantonensis* in toad and frogs in Okinawa Prefecture, Japan. Japan J Parasitol.

[5] Bhaibulaya M (1968). A new species of *Angiostrongylus* in an Australian rat, *Rattus*
*fuscipes*. Parasitology.

[6] Bolt G, Monrad J, Koch J, Jensen AL (1994). Canine angiostrongylosis: a review. Vet Rec.

[7] Box EK, Yabsley MJ, Garrett KB, Thompson AT, Wyckoff ST, Cleveland CA (2021). Susceptibility of anurans, lizards, and fish to infection with *Dracunculus* species larvae and implications for their roles as paratenic hosts. Sci Rep.

[8] Burke JB, Rodgers LJ (1982). Gastric ulceration associated with larval nematodes (*Anisakis* sp. type I) in pen reared green turtles (*Chelonia*
*mydas*) from Torres Strait. J Wildl Dis..

[9] Bursey CR (1986). Histological aspects of natural eustrongyloid infections of the northern water snake, *Nerodia*
*sipedon*. J Wildl Dis.

[10] Colella V, Knaus M, Lai O, Cantile C, Abramo F, Rehbein S (2019). Mice as paratenic hosts of *Aelurostrongylus*
*abstrusus*. Parasit Vectors.

[11] Deslyper G, Doherty DG, Carolan JC, Holland CV (2019). The role of the liver in the migration of parasites of global significance. Parasit Vectors.

[12] Djennoune D, Marniche F, Amroun M, Boulay R (2018). Comparative diet of hedgehogs (*Atelerix*
*algirus*) in two localities in Kabylia, Algeria. Turk J Zool..

[13] Falsone L, Colella V, Napoli E, Brianti E, Otranto D (2017). The cockroach *Periplaneta*
*americana* as a potential paratenic host of the lungworm *Aelurostrongylus*
*abstrusus*. Exp Parasitol.

[14] Foronda P, López-González M, Miquel J, Torres J, Segovia M, Abreu-Acosta N (2010). Finding of *Parastrongylys*
*cantonensis* (Chen, 1935) in *Rattus*
*rattus* in Tenerife, Canary Islands (Spain). Acta Trop.

[15] Fuehrer HP, Morelli S, Bleicher J, Brauchart T, Edler M, Eisschiel N (2020). Detection of *Crenosoma* spp, *Angiostrongylus*
*vasorum* and *Aelurostrongylus*
*abstrusus* in gastropods in eastern Austria. Pathongens..

[16] Gerichter ChB (1949). Studies on the nematodes parasitic in the lungs of *Felidae* in Palestine. Parasitology.

[17] Hidelaratchi MD, Riffsy MT, Wijesekera JC (2005). A case of eosinophilic meningitis following monitor lizard meat consumption, exacerbated by anthelminthics. Ceylon Med J.

[18] Hobmaier M, Hobmaier A (1935). Intermediate hosts of *Aelurostrongylus*
*abstrusus* of the cat. Exp Biol Med.

[19] Izquierdo-Rodriguez E, Martin-Carrillo N, Valladares B, Foronda P (2020). Study of zoonotic enteric pathogens of *Atelerix*
*algirus* in Tenerife, Canary Islands, Spain. Front Vet Sci.

[20] Jeżewski W, Buńkowska-Gawlik K, Hildebrand J, Perec-Matysiak A, Laskowski Z (2013). Intermediate and paratenic hosts in the life cycle of *Aelurostrongylus*
*abstrusus* in natural environment. Vet Parasitol.

[21] Jones HI (1978). Abbreviata (Nematoda: Physalopteroidea) from western Australian snakes. Aust J Zool.

[22] Jones HI (2010). Dwarf monitor lizards (Varanidae: *Varanus*, *Odatria* s. gen.) as definitive and paratenic hosts for physalopteran nematodes. Aust J Zool..

[23] Kanpittaya J, Jitpimolmard S, Tiamkao S, Mairiang E (2000). MR findings of eosinophilic meningoencephalitis attributed to *Angiostrongylus*
*cantonensis*. Am J Neuroradiol.

[24] Kearse M, Moir R, Wilson A, Stones-Havas S, Cheung M, Sturrock S (2012). Geneious basic: an integrated and extendable desktop software platform for the organization and analysis of sequence data. Bioinformatics.

[25] Lai CH, Yen CM, Chin C, Chung HC, Kuo HC, Lin HH (2007). Eosinophilic meningitis caused by *Angiostrongylus*
*cantonensis* after ingestion of raw frogs. Am J Trop Med Hyg.

[26] Lange M, Penagos-Tabares F, Hirzmann J, Failing K, Schaper R, Van der Bourgonie YR (2018). Prevalence of *Angiostrongylus*
*vasorum*, *Aelurostrongylus*
*abstrusus* and *Crenosoma*
*vulpis* larvae in native slug populations in Germany. Vet Parasitol.

[27] Mackerras MJ (1957). Observations on the life history of the cat Lungworm. *Aelurostrongylus*
*abstrusus* (Railliet, 1898) (Nematoda: Metastrongylidae). Aust J Zool..

[28] Magnino S, Colin P, Dei-Cas E, Madsen M, McLauchlin J, Nöckler K (2009). Biological risks associated with consumption of reptile products. Int J Food Microbiol.

[29] Martin-Alonso A, Abreu-Yanes E, Feliu C, Mas-Comas S, Bargues MD, Valladares B (2015). Intermediate hosts of *Angiostrongylus*
*cantonensis* in Tenerife, Spain. PLoS ONE.

[30] Medina M, Nogales M (2009). A review on the impacts of feral cats (*Felis*
*silvestris*
*catus*) in the Canary Islands: implications for the conservation of its endangered fauna. Biodivers Conserv.

[31] Morand S, Bouamer S, Hugot JP (2006). Micromammals and macroparasites: from evolutionary ecology to management.

[32] Mouhoub-Sayah C, Djoudad-Kadji H, Kletty F, Malan A, Robin JP, Saboureau M (2018). Seasonal variations in the diet and food selection of the Algerian hedgehog *Atelerix*
*algirus*. Afr Zool.

[33] Mozzer LR, Lima WS (2015). *Gallus*
*gallus*
*domesticus*: paratenic host of *Angiostrongylus*
*vasorum*. Vet Parasitol.

[34] Panackel C, Vishad CG, Vijayakumar K, Sharma RN (2006). Eosinophilic meningitis due to *Angiostrongylus*
*cantonensis*. Indian J Med Microbiol..

[35] Parker GA, Ball MA, Chubb JC (2015). Evolution of complex life cycles in trophically transmitted helminths. 1. Host incorporation and tropic ascent. J Evol Biol..

[36] Pleguezuelos JM. In: Pleguezuelos JM, Márquez R, Lizana M, eds. Atlas y libro rojo de los anfibios y reptiles de España. Madrid: Dirección General de Conservación de la Naturaleza; 2002.

[37] Pozio E (2005). The broad spectrum of Trichinella hosts: from cold- to warm-blooded animals. Vet Parasitol.

[38] Qvarnstrom Y, da Silva AC, Teem JL, Hollingsworth R, Bishop H, Graeff-Teixeira C (2010). Improved molecular detection of *Angiostrongylus*
*cantonensis* in mollusks and other environmental samples with a species-specific internal transcribed spacer 1-based TaqMan assay. Appl Environ Microbiol.

[39] Rosen L, Laigret J, Bories S (1961). Observation on an outbreak of eosinophilic meningitis on Tahiti, French Polinesia. Am J Epidemiol.

[40] Sánchez Vicente S. Contribución al conocimiento de la parasitofauna (Helmintos y Artrópodos) de mamíferos no lagomorfos de Canarias. Barcelona: Departament de Microbiologia i Parasitologia Sanitàries, Universitat de Barcelona; 2013 (in Spanish).

[41] Segeritz L, Cardona A, Taubert A, Hermosilla C, Ruiz A (2021). Autochthonous *Angiostrongylus*
*cantonensis*, *Angiostrongylus*
*vasorum* and *Aelurostrongylus*
*abstrusus* infections in native terrestrial gastropods from the Macaronesian Archipelago of Spain. Parasitol Res.

[42] Udiz-Rodriguez R, García-Livia K, Valladares-Salmerón M, Dorta-Almenar MN, Martín-Carrillo N, Martin-Alonso A (2018). First ocular report of *Gurltia*
*paralysans* (Wolffhügel, 1933) in cat. Vet Parasitol.

[43] Wallace GD, Rosen L (1986). Studies on eosinophilic meningitis. 2. experimental infection of shrimps and crabs with *Angiostrongylys*
*cantonensis*. Am J Epidemiol..

[44] Wang QP, Wu ZD, Wei J, Owen RL, Lun ZR (2012). Human *Angiostrongylus*
*cantonensis*: an update. Eur J Clin Microbiol Infect Dis.

